# Adjective position in the code-switched speech of Spanish and Papiamento heritage speakers in the Netherlands: Individual differences and methodological considerations

**DOI:** 10.3389/fpsyg.2023.1136023

**Published:** 2023-05-02

**Authors:** Brechje van Osch, M. Carmen Parafita Couto, Ivo Boers, Bo Sterken

**Affiliations:** ^1^Acqva Aurora Center, Department of Language and Culture, UiT, The Arctic University of Norway, Tromsø, Norway; ^2^Heritage Linguistics Lab, Leiden University, Center for Linguistics, Leiden, Netherlands; ^3^Language Variation and Textual Categorization, Faculty of Philology and Translation, University of Vigo, Vigo, Spain; ^4^Department of Netherlandic Studies, Károli Gáspár University, Budapest, Hungary

**Keywords:** code-switching, heritage bilingualism, adjective position, extra-linguistic variables, individual differences, Papiamento, Dutch, Spanish

## Abstract

**Introduction:**

This study examines adjective-noun order in code-switched constructions by heritage speakers of Spanish and Papiamento in the Netherlands. Given that Dutch differs from Spanish and Papiamento regarding the default position of the adjective, word order in the nominal domain creates a so-called “conflict site” in code-switching. Most accounts of word order patterns in code-switching focus on structural constraints, such as the matrix language or the strength of the EPP feature in Agr. Thus far, studies comparing the two models have not found compelling evidence for either of them.

**Methods:**

The present study takes a more comprehensive approach and considers several linguistic (matrix language, adjective language, and type of insertion) as well as extra-linguistic variables (e.g., age, age of onset, and patterns of exposure and use). Moreover, we compare heritage speakers of two different heritage languages that are linguistically similar (both Spanish and Papiamento exhibit postnominal adjectives), and share the same dominant societal language, but are likely to differ from each other in terms of certain sociolinguistic properties. 21 Spanish and 15 Papiamento heritage speakers (aged 7–54) in the Netherlands carried out a Director-Matcher task, aimed at eliciting nominal constructions containing switches.

**Results:**

The results show that either the ML or the language of the adjective, or both, are important predictors for word order, although the data cannot disentangle these two factors. Moreover, the type of insertion was found to play a role: word order patterns for noun insertions differed from other types of insertions. In addition, the two groups did not behave similarly: Papiamento speakers were more categorical in their preference for noun-adjective order when inserting Dutch nouns into their heritage language than the Spanish speakers were. Finally, there was a great deal of individual variation, which seemed to be related mostly to the age of the participants: children and teen participants behaved differently from adults.

**Discussion:**

These findings demonstrate that both linguistic and extra-linguistic play a role in determining how heritage speakers deal with conflict sites in the nominal domain. Particularly, the findings suggest that, at least for some communities and in some code-switching modes, children may need more time, or more input, too converge on adult-like code-switching norms.

## 1. Introduction

Heritage speakers (HSs) are bi/multilingual speakers who, like most other multilinguals, commonly use elements from their languages in the same utterance (either within the same sentence or conversation). This phenomenon is known as code-switching (CS; Deuchar, 2012). In studies of heritage language (HL) acquisition, code-switching has often been overlooked, as the focus of most studies is on either the heritage language of the bilingual or their majority/dominant societal language. However, studying code-switching can make important contributions to our knowledge about heritage speakers’ grammar, since it allows us to uncover patterns in a bilingual’s grammar that remain hidden in the study of unilingual speech alone. In recent decades, a general consensus has emerged that code-switching is rule-governed (*cf.* Parafita Couto et al., 2021 for an overview). Nevertheless, “no clear evidence has emerged concerning the structural regularities that underlie mixed speech across language pairs, or even within the same language pair in different communities” (Parafita Couto et al., 2023). Recent studies suggest that different code-switching strategies may be used between members of the same community (e.g., Boers et al., 2020) and also that there are cross-community differences between communities that share the same language combinations, suggesting that sociolinguistic variables may in some cases override structural constraints. However, to date, we still do not have a clear picture of how the interaction of different linguistic and extralinguistic components shapes code-switching outcomes (Stell and Yakpo, 2015).

In this study, we look at two separate communities of heritage speakers who differ from each other in terms of age (comparing children, teens, and adults) as well as age of onset and patterns of use and exposure, in order to investigate which, if any, of these factors play a role in determining code-switching patterns. We focus on heritage speakers of Spanish and Papiamento who live in the Netherlands, targeting switching where the structures of the two languages differ (conflict sites, *cf.* Vaughan-Evans et al., 2020 for a recent overview). In particular, we address word order in adjective-noun switches. Adjectives are pre-nominal in Dutch and (mostly) post-nominal in Papiamento and Spanish (*cf.* section 2). Hence, Spanish-Dutch and Papiamento-Dutch code-switching between the noun and the adjective could result in four potential noun-adjective combinations (Pap/Span N Dutch Adj, Pap/Span Adj Dutch N, Dutch N Pap/Span Adj, and Dutch Adj Pap/Span N), so the question that arises is whether they are all possible or whether some combinations are disallowed in the bilingual grammars of these speakers. Due to the generally low occurrence of attributive adjectives in production data (*cf.* Parafita Couto and Gullberg, 2019), several studies attempted to unveil the constraints that predict code-switching patterns at this conflict site in different bilingual populations (Spanish-English, Welsh-English, and Papiamento-Dutch) using different methodologies (Parafita Couto et al., 2015, 2017a,b; Voss, 2018; Pablos et al., 2019; Stadthagen-González et al., 2019; Vaughan-Evans et al., 2020, i.a.). Most of these studies evaluated the predictions of two theoretical accounts: the Matrix Language Framework (MLF, Myers-Scotton, 1993) and the Minimalist Program approach (MP, Cantone and MacSwan, 2009), although no clear evidence to favor one model over the other was found. However, these studies provided valuable insight into a general preference for noun-insertions over adjective insertions (*cf.* Vaughan-Evans et al., 2020 for a detailed overview). In the next section, we present a brief description of Papiamentu–Dutch and Spanish-Dutch bilingualism and word order.

## 2. Papiamento-Dutch and Spanish-Dutch bilingualism

### 2.1. The Papiamento and Spanish-speaking communities in the Netherlands

Papiamento is a Portuguese-based creole (re)lexified by Spanish (Jacobs, 2012) spoken in Aruba, Bonaire, and Curaçao (known as the ABC islands, the Caribbean), where it is an official language together with Dutch and English. It is the first language of more than 80% of the population (Kester and Fun, 2012; Jacobs and Muysken, 2019). Papiamento is also spoken by a large part of the 161,265 Antillean migrants who live in the (European) Netherlands [Central Bureau of Statistics (CBS), 2019],^1^ a diverse community ranging from “well-established long-term residents of Antillean origin, students, and young people with little chance of employment and living in poor conditions” (Jacobs and Muysken, 2019). The ABC islands are part of the Kingdom of the Netherlands and have thus been in close contact with Dutch for over three and a half centuries. Because of the extensive historical contact with Dutch and because of wide-spread bilingualism in the country of origin, Papiamento in the Netherlands has been described as post-colonial HL, in a similar situation as Hindi in the United Kingdom (Jacobs and Muysken, 2019). Several studies point to the fact that, despite the importance of Dutch in everyday life, Papiamento dominance can still be found in bilingual populations residing in the Netherlands (Pablos et al., 2019; Suurmeijer et al., 2020), and their attitudes to their HL are positive (Kester and Hortencia 2010; Kester and Fun, 2012). Perhaps related to this, the most common code-switching pattern observed in the available data seems to be that Papiamento is the matrix language and Dutch elements—often nouns—are inserted (Muysken et al., 1996; Parafita Couto and Gullberg, 2019).

According to data from the Central Bureau of Statistics (CBS) in 2019, a total of 130,160 people living in the Netherlands come from Spanish-speaking countries. About a third of the Spanish-speaking population comes from Spain, and many of these migrated to the Netherlands in the 1960 and 1970s as contracted workers. The rest came from a range of Spanish speaking countries in Latin America, where dictatorships and civil wars caused a wave of political refugees during the 1970 and 1980s. More Spanish-speaking people migrated to the Netherlands during the 1990s (mostly from Colombia and the Dominican Republic; van Suchtelen, 2016). In the Netherlands, we do not find tight-knit Spanish-speaking communities such as the ones that exist in certain areas in the United States. People tend to live dispersed across the country, and there is relatively little cohesion among its members (van Osch, 2019). Spanish speakers in the Netherlands are appreciated for their linguistic repertoire, as Spanish enjoys a relatively high prestige (van Osch, 2019). From personal communication with several Spanish heritage speakers who participated in the present as well as other studies, we know that many of them only speak Spanish with their direct family members. Therefore, we may even contend that there is no such thing as a “Spanish-speaking community” in the Netherlands, since the word community in and of itself implies membership of a group that has certain characteristics shared between all members, as well as close connections between those individual members.

We do not know of any studies that have investigated code-switching habits for this particular population. Therefore, we do not know whether there are any directionality asymmetries such as those that have been attested for the Papiamento-speaking community.

### 2.2. Word order in Dutch, Spanish, and Papiamento

Spanish and Papiamento are different from Dutch when it comes to noun-adjective word order. While Dutch requires a pre-nominal position of the adjective (Broekhuis, 2013), as shown in (1), Spanish and Papiamento use post-nominal adjectives, as shown in (2) and (3), even though pre-nominal adjectives are sometimes accepted in both languages (see Kouwenberg and Muysken, 1994 and Castillo, 2022 for Papiamento and García-Bayonas, 2006 for Spanish).

Dutch

(1) een zwarte hamer
   a black hammer
   “a black hammer”

Spanish

(2) un martillo negro
   a hammer black
   “a black hammer”

Papiamento

(3) un martin pretu
   a hammer black
   “a black hammer”

In Spanish, the placement of a number of adjectives with respect to the noun varies depending on the semantic interpretation of the adjective, see examples in (4a) and (4b). Certain adjectives tend to be placed before the noun, such as gran (great) and buen (good), or can only appear before the noun, such as mero (mere). Most adjectives however tend to be placed after the noun, and some are strictly ungrammatical in prenominal position, such as adjectives which indicate nationalities or—important to this study—colors (2).

(4) a.un hombre pobre
   a man poor
   “a poor (poverty-stricken) man”
    b. un pobre hombre
   a poor man
   “a poor (piteous) man”

Papiamento adjectives behave similarly to Spanish ones, and may appear prenominally, which then changes its meaning (Sledge, 2011), “encoding a non-restrictive meaning that departs from the regular denotation” (Castillo, 2022, p. 53). Examples (9a) and (9b) demonstrate how the semantic interpretation of an NP differs with different noun-adjective word orders in Papiamento (just as in 4a and 4b).

(5)  a. homber pober
   man poor
   “poor (poverty-stricken) man”
    b. pober homber
   poor man
   “poor (piteous) man” (Parafita Couto et al., 2017a,b, p. 162)

The stimuli for the current study, however, were designed to elicit color adjectives, which leave no room for interpretation and are always postnominal in both Spanish and Papiamento, and prenominal in Dutch.

## 3. Previous literature on word order in code-switching

### 3.1. Grammatical constraints

Poplack (1980) proposed the equivalence constraint, which states that “[c]ode-switches will tend to occur at points in discourse where juxtaposition of L1 and L2 elements does not violate a syntactic rule of either language, i.e., at points around which the surface structure of the two languages map onto each other” (p. 586). This implies that code-switching conflict sites should not happen, yet examples from spontaneous conversational data show that they do, as illustrated by Parafita Couto and Gullberg (2019) for Papiamento-Dutch. In the example un dushi verblijf “a nice stay,” for example, the Papiamento adjective “dushi” precedes the Dutch noun “verblijf,” contrary to what would be expected in unilingual Papiamento constituent order (Parafita Couto and Gullberg, 2019). Below we provide a brief overview of the predictions of some theoretical models to account for such switches.

According to the Matrix Language Framework (MLF, Myers-Scotton, 1993, 2002), there is an asymmetry between the two languages in code-switched discourse, distinguishing between the ‘matrix language’ (ML), which provides the morphosyntactic frame for the clause, and the ‘embedded language’ (EL), which provides embedded elements. The MLF predicts that both finite verb morphology and word order within a clause will be sourced from the same language (the ML). As such, if the finite verb morphology is from language A, then the prediction would for the relative word order within the adjective-noun phrase to also be from language A.

Another approach, which is grounded in the Minimalist Program (MP), assumes that the features of the lexical items should account for CS/bilingual grammars (MacSwan, 1999). Thus, code-switching data should be explained in the same way we explain monolingual grammars. Regarding adjective-noun order, Cantone and MacSwan (2009) follow proposal of Cinque (1994, 1999, 2005) that adjectives universally precede nouns and that the postnominal position of the adjective in languages like Spanish and Papiamento follows from overt movement of the noun to a position to the left of the adjective, due to a strong EPP feature in Agr in those languages. Thus, they arrive at the descriptive generalization that “while the data remain slightly ambiguous, a relatively clear pattern has emerged in both the survey data and the naturalistic data confirming the general view of previous researchers, namely, that the word order requirements of the language of the adjective determine word order in code-switching in DP-internal contexts” (Cantone and MacSwan, 2009, pp. 266–267). Therefore, the language of the adjective, irrespective of the matrix language, is expected to determine the adjective’s position in code-switched phrases (Cinque, 2005; Cantone and MacSwan, 2009). However, Cantone and MacSwan (2009) did not control for the Matrix Language of the clause, so it is not clear whether these examples could also be explained by the MLF.

Several studies have tried to differentiate between these two models, but no clear conclusion can be drawn (*cf.* Parafita Couto et al., 2021 for an overview). For instance, for the specific case of Papiamento-Dutch mixed nominal constructions, study of Pablos et al. (2019) used event-related brain potentials (ERPs) to measure online comprehension of adjective-noun switching, but leading to null results when trying to disentangle the predictions of the different theoretical models. Similarly, Voss (2018) used comparative judgments and showed that neither of the two theoretical models could fully account for the acceptability of Papiamento-Dutch adjective-noun switches.

### 3.2. Extra-linguistic factors

Whereas previous studies on word order have mainly focused on comparing MLF and MP predictions, the current study takes a different approach, which leaves more room for extra-linguistic variables both at the individual level and at the level of the community (*cf.* Parafita Couto and Gullberg, 2019),

Variation at the individual level has been observed by Boers et al. (2020) and van Osch et al. (2022), who demonstrate that differences between speakers with respect to gender agreement strategies in code-switching are related to differences in dominance, in terms of proficiency, use and exposure. Similarly, Liceras et al. (2008) and Munarriz-Ibarrola et al. (2022) report differences in code-switching patterns between groups of bilinguals that seem to be related to the order of acquisition of the languages in the particular bilingual group.

There is also evidence from a usage-based perspective that suggests that code-switching patterns emerge through their increased use and subsequent entrenchment and such patterns can be community-specific (Backus, 2015; Valdés and Jorge, 2016; Blokzijl et al., 2017; Balam et al., 2020, 2022). It has been demonstrated that community-specific norms exist in certain parts of code-switching grammars, and that bilingual communities of the same language pair do not necessarily converge onto the same code-switching structures (e.g., Balam et al. (2020) for code-switched verbal constructions in Spanish-English bilingual communities or Królikowska et al. (2019) for gender assignment to English noun insertions in different Spanish-English communities). Such norms may depend on the frequency of code-switching within the community (Królikowska et al., 2019). It is hence expected that cross-community variation may also affect environments about which the MP or MLF make predictions, such as adjective position, though these models do not account for this type of variation.

An interesting case of cross-community variation that may be relevant to the present topic of investigation concerns code-switching directionality or choice of matrix language. Several studies presenting natural production data show that, within specific communities, speakers tend to converge on one matrix or base language, inserting elements from the other language (e.g., Welsh for Welsh-English in northern Wales, Spanish for Spanish-English in Miami, English Creole for English Creole-Spanish in Nicaragua, Frisian for Frisian-Dutch in the Netherlands, *cf.* Breuker, 2001; Blokzijl et al., 2017; Bosma and Blom, 2019). As mentioned in section 2, a similar asymmetry has also been reported for Papiamento, such that it is more common to insert Dutch elements (such as nouns) into Papiamento, than vice versa (Muysken et al., 1996; Parafita Couto and Gullberg, 2019).

What determines the choice of matrix language is not clear, but previous research indicates that extralinguistic factors such as language prestige play a role (Blokzijl et al., 2017; Parafita Couto and Gullberg, 2019), suggesting that the language with the higher social status is the one that is inserted into the other (matrix) language. These findings highlight the extent to which code-switching practices are embedded in the sociocultural and sociohistorical experiences of the bilingual speakers (*cf.* Suurmeijer et al., 2020) and raise the question of whether exposure to asymmetries in the choice of matrix language or directionality of switching within the community would determine how speakers tackle code-switches at conflict sites such as the one reported on in the present paper. This issue is discussed by Vaughan-Evans et al. (2020), who looked at the relative order of adjectives and nouns in switched nominal constructions Welsh-English by means of an electrophysiological study. They observe stronger expectations about the placement of the code-switch when the ML is Welsh, than when the ML is English, which they attribute to the fact that in this particular community, English insertions into Welsh are considerably more common than vice versa. They argue that this finding could also explain some of the conflicting patterns observed in previous electrophysiological studies (Parafita Couto et al., 2017a,b on Welsh-English and Pablos et al., 2019 on Papiamento-Dutch), which did not consider the frequency of the ML of the sentence as a confounding factor within their experimental design and analyses.

Finally, some studies have observed differences in code-switching patterns between child and adult bilinguals of the same language combination. For instance, Urbaneja (2020) showed that Spanish-English child bilinguals produced more English determiners than adult bilinguals, although not from the same community. Similarly, longitudinal study of Vihman (2018) two English-Estonian bilingual children (aged 2;10–7;2 and 6;6–11;0) shows the importance of considering age as a factor affecting code-switching patterns, as the grammar of the children in the study contains a lot of variation. They have not yet fully acquired adult grammar and therefore do not conform to the constraints of the MLF model, like adult bilinguals. This suggests that, as is the case for the development of unilingual grammars in language acquisition, children’s code-switching patterns and strategies may exhibit more flexibility and take time to converge onto adult-like norms. On the other hand, Balam et al. (2021) and Phillips and Deuchar (2021), who compared children and adults from the same community with respect to gender and choice of the matrix language respectively, do not observe any differences between the different age groups in their studies. The children in the study of Phillips and Deuchar (2021) were aged between 1;9 and 2;6, leading them to conclude that the code-switching patterns in the linguistic input in the community begin to be reproduced in child productions from a very young age.

## 4. Research questions

In the present study, we focus on adjective-noun code-switched constructions in Spanish-Dutch and Papiamento-Dutch bilinguals, and we aim to unveil the factors that determine which word order is preferred by heritage speakers from these languages. To this end, the following research questions were formulated:

1. Which linguistic factors (e.g., the ML and the language of the adjective) determine word order preferences?
2. What is the role of extralinguistic factors, both at the individual level and at the community level, in accounting for heritage speakers’ preferences in code-switched speech?

## 5. Materials and methods

### 5.1. Participants

A total of 36 heritage speakers living in the Netherlands participated in this study. We would like to note that we use the term heritage speaker, even though not all participants are considered as such under all definitions, for example because they arrived in the Netherlands well after the onset of school. However, given that age of onset was one of our variables of interest, it was considered important that our sample included a wide range of ages of onset. Of the 21 Spanish heritage speakers, 11 participants were born in the Netherlands (two of whom spent a few years of their lives in another Spanish-speaking country later in childhood), four arrived in the Netherlands before starting their primary education, and the remaining six arrived in the Netherlands between the ages of 6 and 12. Of the 15 Papiamento heritage speakers, three were born in the Netherlands, two arrived before going to primary school, and the remaining 10 arrived when they were between 6 and 21 years old. However, it must be noted that all Papiamento-speaking participants were exposed to Dutch to a certain extent before arriving in the Netherlands, given that Dutch is an official language in Aruba and Curaçao, where all participants were from. As mentioned, our participants varied considerably regarding their ages at testing (8–54). The participants can be divided into three age groups: children (age 7–12, *n* = 12), teenagers (age 13–18, *n* = 7), and adults (*n* = 17). The Spanish-speaking participants had backgrounds from a range of Spanish speaking countries, such as Argentina, Colombia, Ecuador, Mexico, Paraguay, Peru, and Spain among others. The Papiamento heritage speakers all came from or have a family background in Curaçao and Aruba. The background questionnaire contained several questions about the participants’ patterns of use and exposure to both languages. They were asked to report their usage of Dutch and of the HL, both with immediate family and non-immediate family, the number of hours per week they received other input (which refers to media such as music, books, television, and social media) in their HL, the frequency with which they visited their country of origin [on a scale from 1 (never) to 4 (once or multiple times a year)], their self-reported skill in their HL (on a scale from 0 to 3 for reading, writing, speaking, and listening separately), and whether they had received any official classes/courses in their heritage language while living in the Netherlands. The questionnaire for the children also contained questions about current input and input in their heritage language at age 0–4. This information is summarized in Table 1 below.

**Table 1:** Socio-linguistic information about the participants.

	Spanish (*N* = 21)	Papiamento (*N* = 15)
Age at testing	M: 17,19	M: 27,27
	Range: 8–52	Range: 9–54
Age of arrival	M: 3,23	M: 8,26
	Range: 0–12	Range: 0–21
Length of residence	M: 13,62	M: 18,87
	Range: 4–37	Range: 3–42
Self-reported skill across domains in the HL (0–3)	M: 2,42	M: 2,13
	Range: 1–3	Range: 0.5–3
Heritage language usage immediate family	M: 47,61%	M: 49,35%
	Range: 10–100	Range: 9–100
Heritage language usage non-immediate family	M: 27,43%	M: 23,31%
	Range: 0–91	Range: 0–90
Usage of Dutch immediate family	M: 48,82%	M: 44,98%
	Range: 10–100	Range: 9–100
Usage of Dutch non-immediate family	M: 68,66%	M: 71,73%
	Range: 5–100	Range: 40–100
Other input in HL (hours a week)	M: 12,1	M: 8,18
	Range: 0–33	Range: 0–67
Heritage language classes (yes/no)	May-21	0/15
(Children) Current input HL	M: 54,63%	M: 45,6%
	Range: 40–100	Range: 24–85
(Children) Current input Dutch	M: 38,44%	M: 43,10%
	Range: 0–55	Range: 15–75
(Children) Previous input HL (0–4 years old)	M: 60,34%	M: 71,9%
	Range: 47,5–100	Range: 46,5–80
(Children) Previous input Dutch (0–4 years old)	M: 33,13%	M: 19,6%
	Range: 0–52,5	Range: 11–25

### 5.2. Materials

The participants completed a Director-Matcher task (*cf.* Gullberg et al., 2009), a method used to elicit nominal constructions consisting of a determiner, noun and adjective (e.g., “above the green painting is a blue lamp”). This task, which has been used before by Bellamy et al. (2018) and Munarriz Ibarrola et al. (2022), consists of a board game involving two people; the director and the matcher. The participants sit across from each other with a cardboard box dividing them, so that they cannot see each other’s board. Both participants have a set of cards laid out depicting different objects in different colors. The goal is for the director to communicate to the matcher where to put the cards, describing the images on each card. If the game is played correctly, both the director and the matcher end up having their cards in the same order on their boards. Both the director and the matcher were given the same set of 30 cards depicting 15 different highly frequent objects (a house, a hat, a bed, etc.) in four different colors: red, white, black and green.

As described in the participants’ section, the background questionnaire was mainly aimed at participants’ current use and exposure to both languages, their education in the HL, and their self-rated proficiency in the HL. The background questionnaire for participants under the age of 12 was filled out by the parents, and also contained a part on the age, education and language use of the father and mother, as well as questions about previous input.

Both the materials and the language background questionnaire can be found on: https://osf.io/3srzv/?view_only=a38aceb650a04dbd8eeff1c84ea867c0

### 5.3. Procedure

The participants completed the task four times in total. Examples 6–9 show samples in the four modes of a Spanish HS. The order of administration was as follows: the first two modes elicited nominal constructions in the two languages in unilingual mode [first the HL, then Dutch—examples (6) and (7)], in order to check whether the participants were able to use the target word order in each of their languages. Immediately after the unilingual modes, they carried out the same task in two different code-switching modes. First, they were instructed to complete the task in their HL again, but this time they were asked to name just the object in Dutch (8). Finally, the participants were instructed to use Dutch, and name the object in the heritage language (9). This order was chosen for two reasons. Based on the assumption that our participants were not likely to highly frequent code-switchers, we considered that it would be easier for them to understand the task if they started with the unilingual mode. Moreover, maintaining the same order for every participant allowed us to analyze observed differences between groups without having to take into account any potential effect of order.

(6) Arriba de la casa roja está el libro blanco
   Above de house red is the book white
   “Above the red house is the white book”
(7) Naast de zwarte kam ligt de groene hoed
   Next to the black comb is the green hat
   “Next to the black comb is the green hat”
(8) *A la derecha de la* **bloem** *blanca está el* **boek** *verde*^2^
   To the right of the flower white is the book green
   “To the right of the white flower is the green book”
(9) *Onder het zwarte* **casa** is *de rode* **flor**
   Underneath the black house is the red flower
   “Underneath the black house is the red flower”

At the beginning of the procedure, the participants were asked in which language they would like to receive instructions, the questionnaire, and consent forms, in Dutch or in their heritage language. The participants (or their parents in the case of child participants) first signed a consent form. After this, they completed the task while being given precise instructions. Only after they had completed the first round of the task in the heritage language were they told to do the next round in Dutch, and so on. After having completed all four rounds of the task, the participants (and/or parents) were asked to fill out the background questionnaire.

## 6. Analysis and results

In the analysis presented below, we only included those instances where an adjective was produced either directly preceding or following the noun. Those cases that lacked an adjective (*n* = 39) or where the adjective was part of a relative clause construction (*n* = 29; *een hoed* **que es verde**—“a hat that is green”) were excluded.

### 6.1. Unilingual mode

Table 2 shows the frequencies of the produced word orders by both groups combined in the unilingual modes. In the Dutch mode, participants produced almost exclusively adjective-noun word order, except for five instances of noun-adjective order, four of which were produced by the same participant, a Spanish heritage speaker. In the unilingual HL mode, there were 27 occurrences of adjective noun orders, 23 of which were produced by the same participant, a Papiamento HS.

**Table 2:** Produced word order in the unilingual experimental modes.

	Adjective-Noun	Noun-Adjective
Dutch mode	1,186	5
HL mode	27	1,108

### 6.2. Code-switching mode

In code-switching mode, HSs tended to adhere to the word order from the experimental mode they were in, that is: they used prenominal adjectives more when they were instructed to speak Dutch with nouns inserted from the HL and they produced postnominal adjectives more when they had to insert Dutch nouns into their respective heritage languages (see Table 3). However, there is variation: in the Dutch mode with HL insertions, 278 (24,11%) of all inserted nouns have a postnominal adjective, and in the HL mode with Dutch insertions, adjective-noun order was used 130 (11,31%) times.

**Table 3:** Produced word order in the code-switching experimental modes.

	Adjective-Noun	Noun-Adjective
Dutch mode with HL insertions	875	278
HL mode with Dutch insertions	130	1,019

#### 6.2.1. Linguistic variables

In this section, we ask to what extent this variability can be explained by linguistic factors. In this part of the analysis, we collapse the data for the two heritage groups, given that Spanish and Papiamento behave similarly when it comes to word order in the nominal domain. In the introduction, two linguistic variables were mentioned that have been proposed to account for word order constraints in code-switching: the matrix language and the language of the adjective. While identifying the language of the adjective is straight-forward, the same is not true when it comes to determining the matrix language. Even though the participants were instructed to speak one language and embed nouns from the other language, it is not guaranteed that they in fact consistently follow these instructions. A potential solution to this problem is to determine the matrix language for each clause based on the language of the verb (*cf.* Herring et al., 2010; Blokzijl et al., 2017; Urbaneja, 2020). However, in our dataset, only 51.6% of utterances included a verb. Of the sentences that lacked a verb, there were sometimes other elements, such as adverbs and/or conjunctions (*en daarnaast weer een zwarte* casa—“and next to that again a black house”). In 99% of these cases, the languages of the verb or these other elements coincided with the language of the experimental mode. Based on this information, it was considered safe to assume that the language of the verb and/or other elements in the sentence could be used as an indicator for the matrix language.

A total of 711 instances that consisted of *only* noun phrases were excluded, leaving us with 1,574 instances. Table 4 presents the word orders produced for these 1,574 cases, by matrix language and adjective language.

**Table 4:** Production of word orders by matrix language and adjective language.

Matrix language	Language adjective	Adjective-Noun	Noun-Adjective
Dutch	Dutch	607	152
	Spanish/Papiamento	0	22
Spanish/Papiamento	Spanish/Papiamento	38	712
	Dutch	7	36

What immediately becomes clear from this table, is that the matrix language almost always coincides with the language of the adjective (1,509 out of 1,574–95,9%). This could be due to the nature of our task: participants were explicitly instructed to name *only* the object in the other language. This led to a high number of noun insertions [example (10); *n* = 1,441].

When the matrix language and the adjective were Dutch, adjective-noun (the Dutch word order) was used more often (607 out of 759 cases), whereas when the matrix language and the adjective were Spanish/Papiamento, noun-adjective (the Spanish/Papiamento word order) was preferred (712 out of 750 cases). For the few cases where the language of the adjective did not coincide with the matrix language (65 in total), we see a general preference for noun-adjective word order, which sometimes aligned with the matrix language (*n* = 36) and other times language of the adjective (*n* = 22). These data thus suggest that either the matrix language or the language of the adjective, or both, seem to play a role in determining word order in code-switched productions. However, the data cannot help us disentangle between these two factors. Moreover, even when both the matrix language and the language of the adjective align, there is still variation, which suggests there may be other factors playing a role.

Taking a closer look at our data, we noticed that the type of insertion mattered. In addition to the 1,441 noun insertions (example 10), there were also 66 determiner-noun insertions (example 11), 18 adjective insertions (example 12), 30 noun + adjective insertions (example 13), and 18 det + noun + adjective insertions (example 14).^3^

(10) “*El* **kam** *negro está arriba*” (Spanish ML, Dutch insertion)
   The comb black is above
   “The black comb is above”
(11) “*Después es* **de bloem** *negro*”(Spanish ML, Dutch insertion)
   Next is the flower black
   “Next is the black flower”
(12) “…*en een bloem* **blanku**”(Dutch ML, Papiamento insertion)
   and a flower white
   “… and a white flower”
(13) “…*met daaronder een* **kama pretu**”(Dutch ML, Papiamento insertion)
   With underneath a bed black
   “…with underneath a black bed”
(14) “*Daarna* **un llave rojo**”(Dutch ML, Spanish insertion)
   after that a key red
   “After that, a red key”

Table 5 below shows the word order preference for each type of insertion that was observed in the dataset.

**Table 5:** Production of word order by matrix language and type of insertion.

Matrix Language	Type of insertion	Adjective-Noun	Noun-Adjective
Dutch	Noun	605 (*het rode* **casa**)	115 (*het* **casa** *rood*)
	Determiner + noun	0 (**la** *rode* **casa**)	37 (***la casa** rood*)
	Determiner + noun + adjective	0 (**la roja casa**)	4 (**la casa roja**)
	Noun + adjective	0 (*het* **roja casa**)	7 (*het* **casa roja**)
	Adjective	5 (*het* **roja** *huis*)	2 (*het huis* **roja**)
Spanish/Papiamento	Noun	38 (*la roja* **huis**)	683 (*la* **huis** *roja*)
	Determiner + noun	0 (**het** *roja* **huis**)	29 (**het huis** *roja*)
	Determiner + noun + adjective	1 (**het rode huis**)	13 (**het huis rood**)
	Noun + adjective	2 (l*a* **rode huis**)	21 (*la* **huis rood**)
	Adjective	0 (*la* **rode** *casa*)	11 (*la casa* **rood**)

Bold type is used to indicate the inserted elements, while italics are used to indicate the matrix language.

What becomes clear from Table 5 is that, apart from noun insertions, all other types of insertions seem to favor noun-adjective order, regardless of the ML.

To see whether any of these effects was statistically significant, we ran a series of linear mixed effects regression models, using the lme4 package in R (R Core Team, 2021). The dependent variable was word order (adjective-noun vs. noun-adjective). Our three predictor variables of interest were matrix language (Dutch vs. HL), adjective language (Dutch vs. HL), and insertion type (noun insertion vs. other insertion), which were all sum-coded. It was problematic to include all three independent variables in a single analysis, for two reasons. First, as explained above, there was a considerable overlap between the matrix language and the language of the adjective: these two factors overlapped for 96% of the data. In addition, the third variable, insertion type, is partially derived from the other two variables, because if the ML and the language of the adjective do not coincide, this automatically implies that the insertion contains at least the adjective, whereas if they do coincide, the insertion can only contain the determiner and/or the noun, but not the adjective. To avoid issues with multicollinearity, we therefore decided to first run three models for each of the three variables separately, and check which of the variables explained the most variance. Each of these models was compared to a null model, i.e., a model only containing the intercept and the random intercept for subject. All three variables improved the model fit significantly, but the model including matrix language showed the most improvement, in terms of both the Akaike Information Criterion (AIK) and the Bayesian Information Criterion (BIC). In the next step we added first the main effect of insertion type and then the interaction between the two variables, and both improved the model significantly.

**Figure 1:**
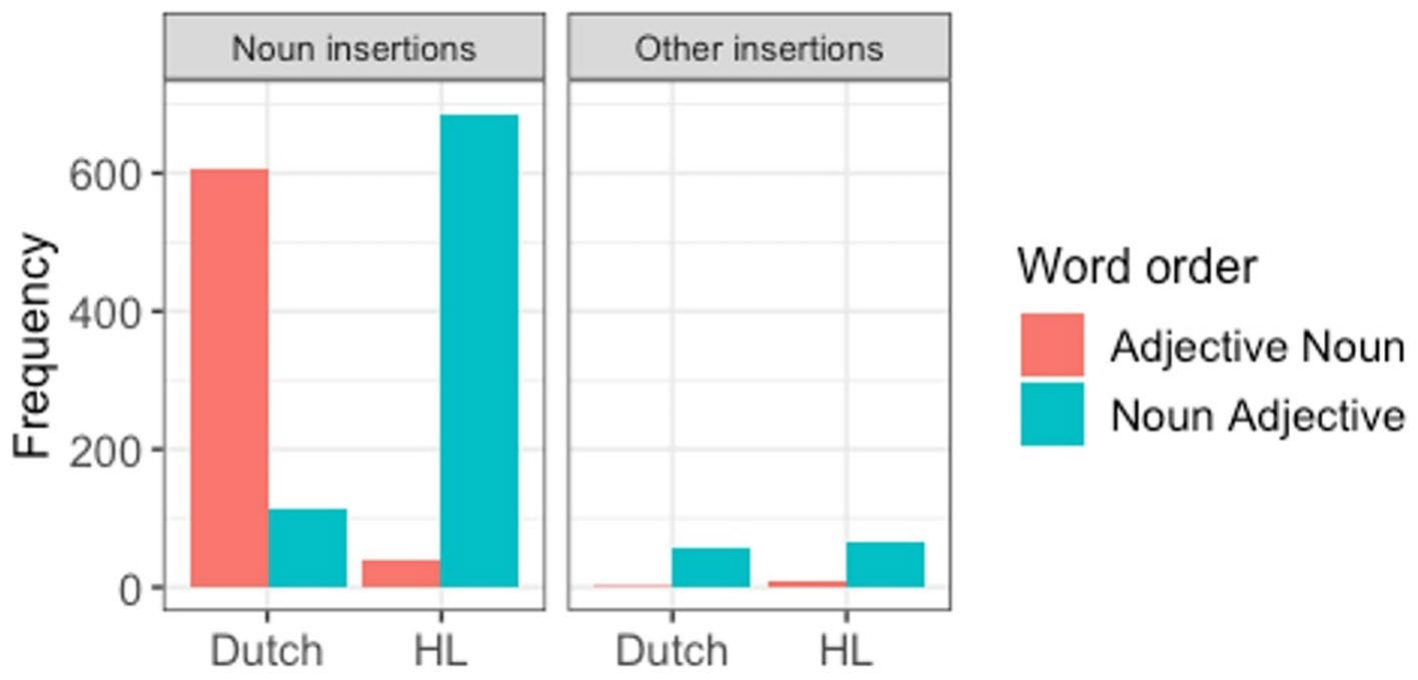
Production of word order in code-switching mode by ML and insertion type.

The final model (Table 6), which also included the random slope for matrix language (the model did not reach convergence when we added the slope for the interaction), showed strong and significant effects for matrix language (β = 9.4, SE = 3.17, *z* = 2.97, *p* = 0.003), insertion type (β = 9.66, SE = 2.74, *z* = 3.52, *p* < 0.001), and the interaction between these two variables (β = −22.60, SE = 5.48, *z* = −4.12, *p* < 0.001), which confirmed the observation that the word order is determined by the Matrix language in the case of noun insertions, but not for all other types of insertions, in which case noun-adjective is the preferred word order overall (Figure 1).^4^

**Table 6:** Output for the final model including linguistic variables.

*Predictors*	Word_order				
	*Estimate*	*Std. error*	*CI*	*Statistic*	*p*
(Intercept)	4.35	1.57	1.28–7.42	2.77	**0.006**
ML based verb or other elements SPAPAP merged 1	9.40	3.17	3.20–15.61	2.97	**0.003**
Noun vs. other based on verb other elements 1	9.66	2.74	4.28–15.04	3.52	**<0.001**
ML based verb or other elements SPAPAP merged 1 × noun vs. other based on verb other elements 1	−22.60	5.48	−33.34–−11.86	−4.12	**<0.001**
Random effects					
σ^2^	3.29				
τ_00 Subject_	74.33				
τ_11 Subject.ML_based_verb_or_otherelements_SPAPAPmerged1_	259.00				
ρ_01 Subject_	−0.43				
ICC	0.98				
*N*_Subject_	31				
Observations	1,574				
Marginal *R*^2^/Conditional *R*^2^	0.426/0.987				

Statistically significant *p*-values are indicated in bold type.

#### 6.2.2. Extra-linguistic variables

In addition to the linguistic variables discussed in the previous section, we were also interested to what extent extra-linguistic variables played a role in determining word order variation. This is why we collected data from two different communities of heritage speakers in the Netherlands, and we also included a wide range of speakers of different ages, different lengths of residence, etc. In this section, we focus on the code-switching data, because both groups were very categorical in the unilingual modes.

**Figure 2:**
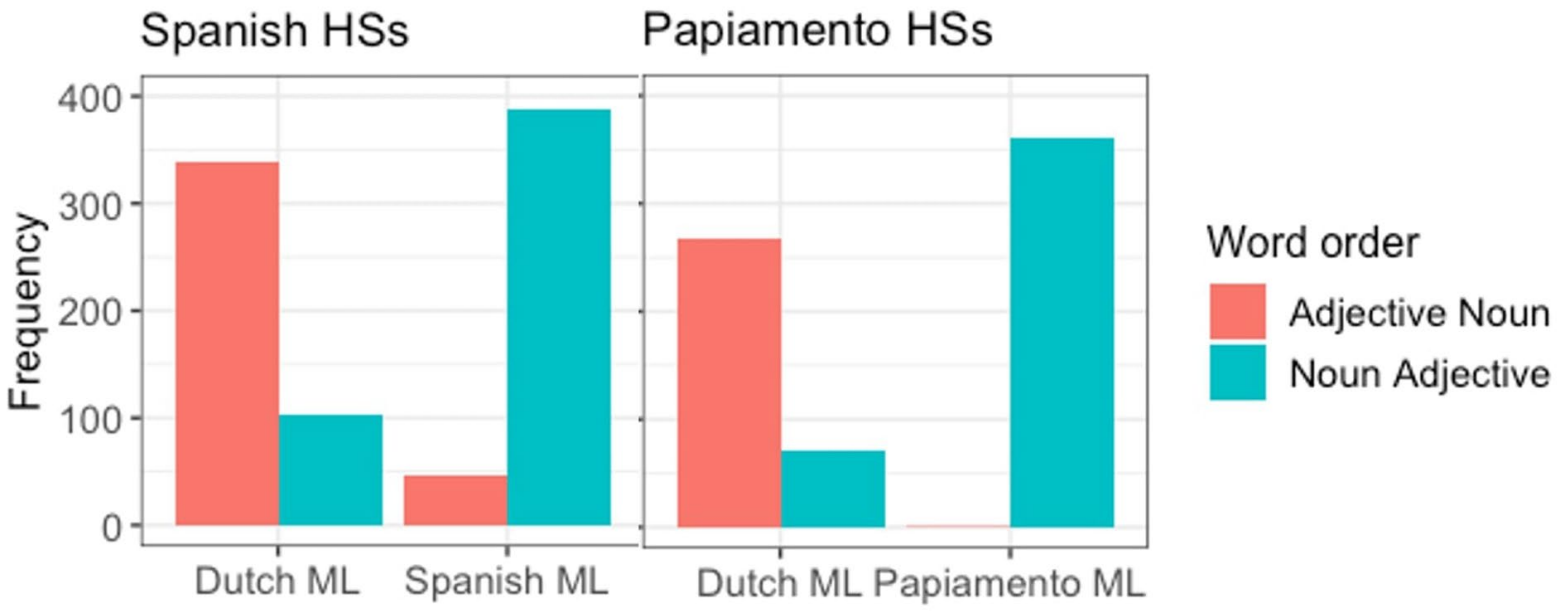
Production of word order by ML, separated between groups.

First, we compare the two communities to each other (Figure 2). While the Spanish HSs show variation in terms of their word order preferences both when the matrix language is Dutch and when it is Spanish, the Papiamento speakers very categorically choose noun-adjective when Papiamento is the matrix language.

**Figure 3:**
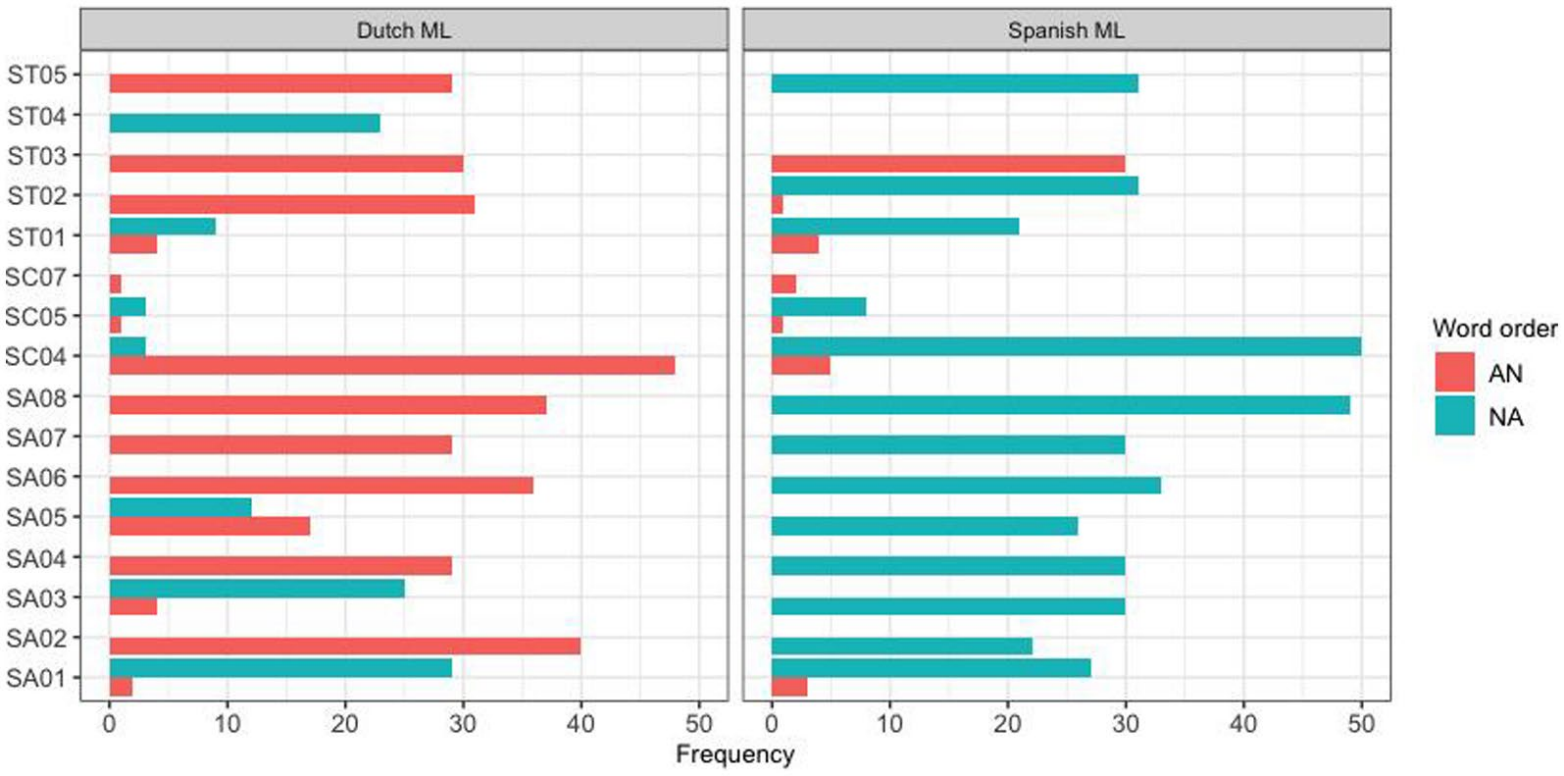
Word order production pattern for individual Spanish HSs, separated by the ML.

**Figure 4:**
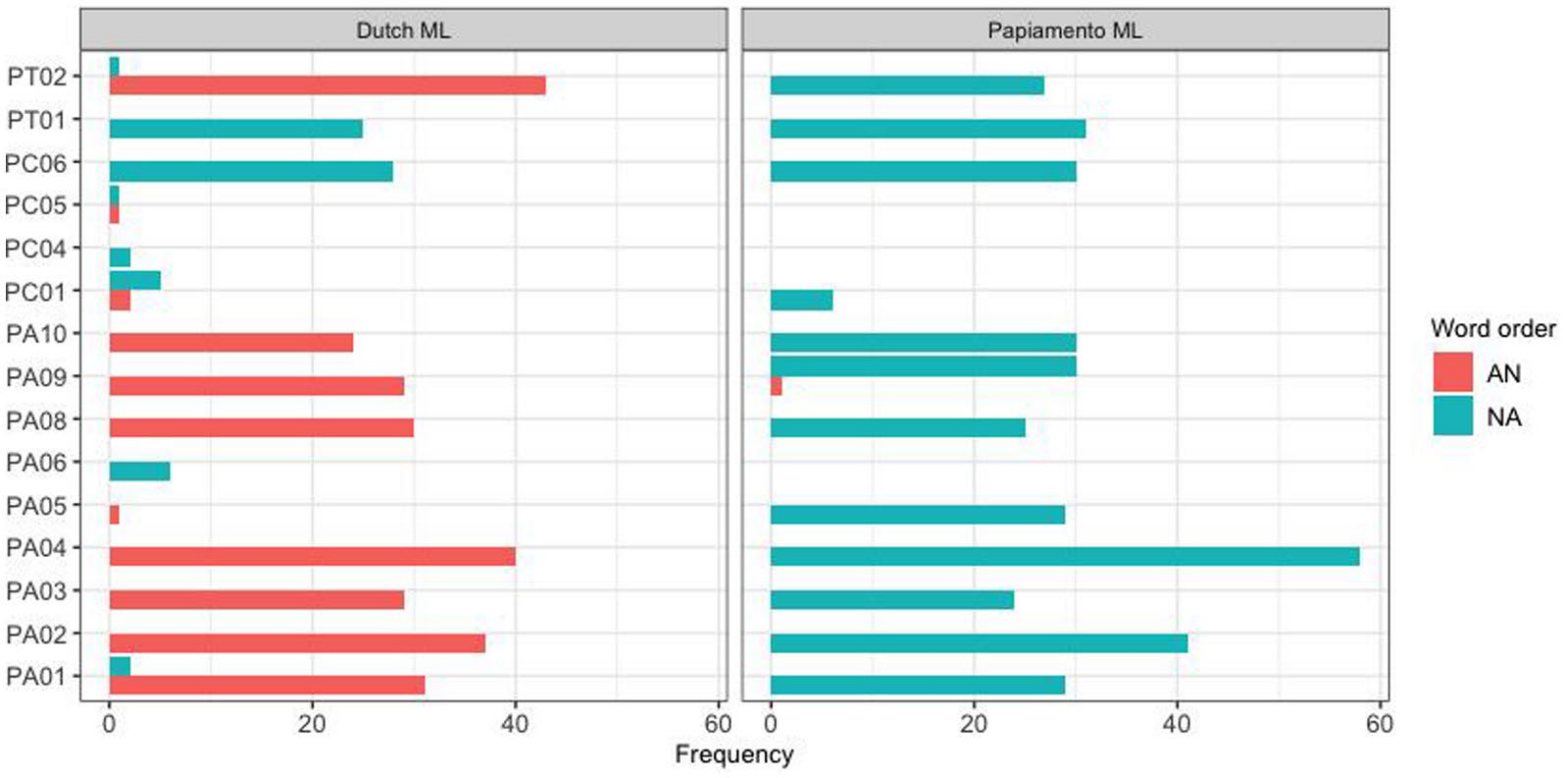
Word order production pattern for individual Papiamento HSs, separated by the ML.

In addition to the difference between these two communities, a large part of the observed variation was found to derive from individual variation between subjects. This is illustrated in Figures 3, 4 for the Spanish group mode and the Papiamento group, respectively.

In a second analysis, we explored which socio-linguistic variables, if any, could account for the observed variation between participants. From the background questionnaire, we had gathered information about the participants concerning their age at testing, age of onset of the societal language, the length of residence in the Netherlands, the amount of use of both languages with their immediate family and in other contexts, the amount of “other” exposure to their HL through TV, music, reading and social media, and their self-rated proficiency in their HL (averaged across four domains; reading, writing, listening, and speaking).

We performed two analyses, one on the Dutch mode for both groups, and one on the HL mode for the Spanish group only, given that there was close to zero variation in the Papiamento group in this mode. For the analysis on the Dutch ML experimental mode, the dependent variable was word order (adjective-noun vs. noun-adjective). We considered the following predictor variables: heritage community, age at testing, age of onset of the societal language, length of residence in the Netherlands, use of Dutch with immediate family, use of Dutch with non-immediate family, total use of Dutch, average “other” exposure to the HL (i.e., through books, music, TV, and social media), whether or not they had had any instruction in their HL, and self-rated proficiency in their HL. Heritage community was a binary variable with two levels: Spanish and Papiamento. Similarly, instruction in the HL was a binary variable with two levels: yes and no. For these two binary variables, sum-coding was used. Age at testing was a categorical variable with three levels (children, teens, and adults), for which orthogonal sum-to-zero coding was used such that contrast 1 compared teens and children (+1/3 for both) to adults (−2/3) and contrast 2 compared teens (+0.5) to children (−0.5). The remaining predictors of interest were continuous variables which were centered and standardized.

Some of these variables are inherently related. For instance, age at testing, age of onset of the societal language, and length of residence are all derived from one another. To avoid multicollinearity issues, we first checked for each of them to what extent they improved the model fit compared to a null model which just included a random intercept for subject. The variable that explained most variability was age at testing. In a similar way, it was decided to include both usage of Dutch with immediate family and with non-immediate family, but not total usage of Dutch.

**Figure 5:**
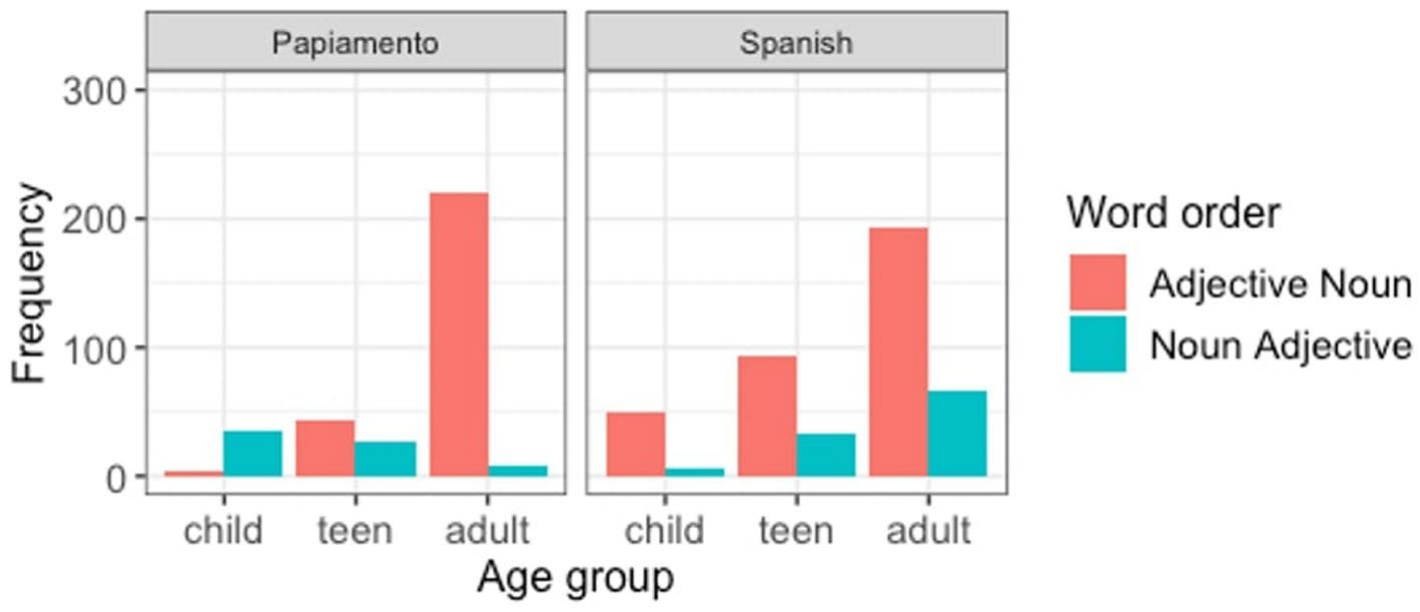
Word order preference for Dutch as a matrix language, by age group by heritage community.

We used the package buildmer (Voeten, 2021) for automatic model selection. The advantage of this package is that it first identifies the maximal model that converges with the variables of interest, and subsequently uses this as a baseline for backward stepwise elimination. However, it does not check multicollinearity for each of the possible models. Therefore, to determine the degree of the correlation between predictor variables, we checked the variance inflation factors (*VIF*) for the final model, and eliminated several variables based on this information. The final model (Table 7) contained significant effects for age group, for the contrast between children and teens vs. adults (β = 10.21, SE = 3.13, *z* = 3.26, *p* = 0.001), heritage community (β = −9.66, SE = 4.01, *z* = −2.41, *p* = 0.016), as well as a significant interaction between these two (β = −15.51, SE = 6.30, *z* = −2.46, *p* = 0.014), which indicated for the Papiamento HSs, younger participants use noun-adjective order relatively more when they insert HL nouns into Dutch, whereas for the Spanish HSs, this is not the case (Figure 5).

**Table 7:** Output for the final model containing extra-linguistic variables in Dutch mode with HL insertions.

*Predictors*	Word_order				
	*Estimate*	*Std. error*	*CI*	*Statistic*	*p*
(Intercept)	−3.07	1.51	−6.03 to −0.11	−2.03	**0.042**
HL1	−9.66	4.01	−17.51 to −1.81	−2.41	**0.016**
Age group1	10.21	3.13	4.08–16.34	3.26	**0.001**
Age group2	−1.86	4.24	−10.17 to 6.44	−0.44	0.660
HL1 × age group1	−15.51	6.30	−27.86 to −3.16	−2.46	**0.014**
HL1 × age group2	−3.24	8.52	−19.95 to 13.46	−0.38	0.704
Random effects					
σ^2^	3.29				
τ_00 Subject_	94.76				
ICC	0.97				
*N*_Subject_	31				
Observations	779				
Marginal *R*^2^/Conditional *R*^2^	0.269/0.975				

Statistically significant *p*-values are indicated in bold type.

**Figure 6:**
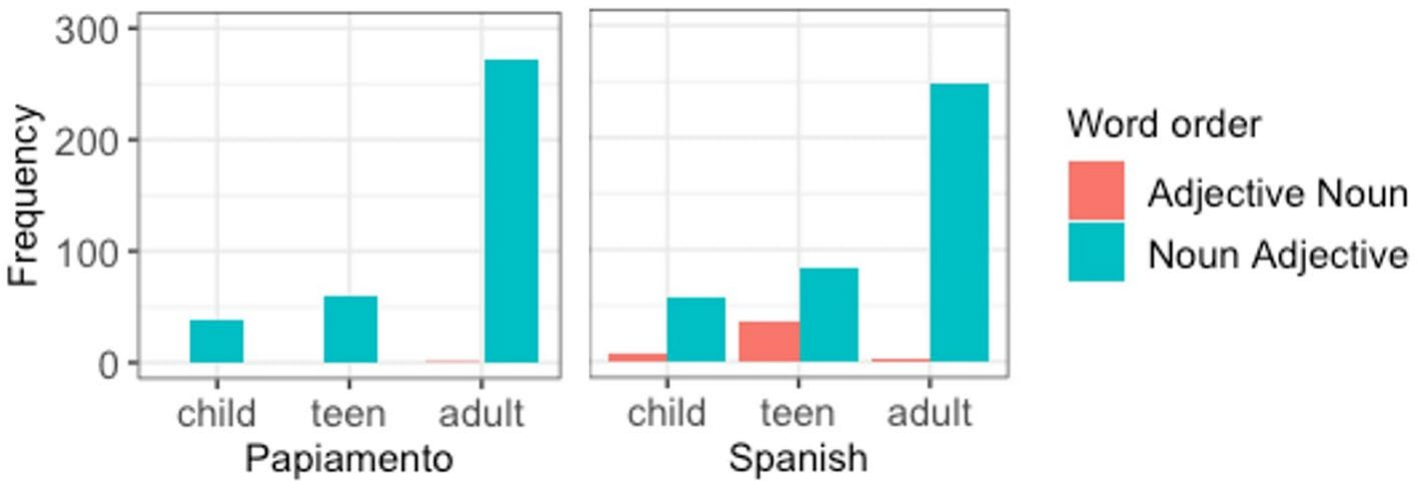
Word order preference for Spanish/Papiamento as a matrix language, by age group by heritage community.

For the analysis on the Spanish experimental mode, the dependent variable was again word order (adjective-noun vs. noun-adjective). For the independent variables, the following were considered: age at testing, age of onset of the societal language, length of residence in the Netherlands, usage of Spanish with the immediate family, usage of Spanish with non-immediate family, total usage of Spanish, exposure to “other” exposure to Spanish (i.e., through books, music, TV, and social media), self-rated proficiency in Spanish, and whether or not they had received instruction in Spanish. Similar to the model for the Dutch experimental mode, instruction in the HL was a binary variable which was sum-coded, age at testing was a ternary variable for which orthogonal sum-to-zero coding was applied as described above, and all other variables were continuous and were centered and standardized.

Through a similar procedure as described above, age at testing was selected over age of onset and length of residence, and usage of Spanish with both immediate and non-immediate family were selected over total usage of Dutch. The final model (Table 8) contained one significant effect of age (β = 8.77, SE = 4.34, *z* = 2.02, *p* = 0.04), as well as a significant intercept for subject. The effect of age indicates that younger participants use the adjective-noun orders relatively more when they insert Dutch nouns into Spanish (Figure 6, right panel). As mentioned earlier, Papiamento speakers of all age groups categorically produced noun-adjective order while inserting Dutch nouns into their HL (Figure 6, left panel).

**Table 8:** Output for the final model containing extra-linguistic variables in HL mode with Dutch insertions.

*Predictors*	Word_order				
	*Estimate*	*Std. error*	*CI*	*Statistic*	*p*
(Intercept)	7.98	2.79	2.50–13.45	2.86	**0.004**
age	8.77	4.34	0.25–17.28	2.02	**0.044**
Random effects					
σ^2^	3.29				
τ_00 Subject_	16.47				
ICC	0.83				
*N*_Subject_	15				
Observations	434				
Marginal *R*^2^/Conditional *R*^2^	0.795/0.966				

Statistically significant *p*-values are indicated in bold type.

## 7. Discussion

The study presented in this paper was concerned with the investigation of word order in the nominal domain in both unilingual and code-switched speech of bilingual speakers of Dutch (a language that has prenominal adjectives) and Spanish or Papiamento (in which adjective are typically placed in the postnominal position). We observed that word order in these cases is constrained both by linguistic factors and by non-linguistic factors.

Concerning linguistic factors, similar to previous studies (Voss, 2018; Stadthagen-González et al., 2019; Vaughan-Evans et al., 2020) we found effects of the matrix language and the language of the adjective. When both the matrix language and the adjective were in Dutch, the preferred order was adjective-noun, and when the matrix language and the adjective were in Spanish or Papiamento, noun-adjective was the preferred order. These findings may indicate support for the role of the Matrix Language Framework (*cf.* Myers-Scotton, 2002). However, it may also be the language of the adjective (or the strength of the EPP feature in AGR, *cf.* Cantone and MacSwan, 2009) that is responsible for the patterns we observe. It is worth noting, however, that almost all switches that adhered to the predictions of both the Matrix Language Frame and the MP included a noun insertion (which are frequent in naturalistic production, Muysken et al., 1996; Parafita Couto and Gullberg, 2019). Like previous studies (Parafita Couto et al., 2015; Voss, 2018; Pablos et al., 2019; Parafita Couto and Gullberg, 2019; Stadthagen-González et al., 2019 among others), our data do not allow us to differentiate between the matrix language and the language of the adjective. Most of the data either are in line with the predictions of both these theories, or they contradict both theories, or they support either of the two. Nonetheless, a novel finding in the present study is the relation between the type of insertion and word order. We noted that noun insertions behaved differently from all other types of insertions, that is: for noun insertions, the above mentioned effects of the matrix language and/or the language of the adjective apply, but for all other types of insertions, noun-adjective was the preferred option across the board. How can we explain this?

Let us start with the second most frequently produced type of insertion after noun insertions: determiner-noun insertions, illustrated in example 15 for Spanish with a Dutch insertion (repeated here) and 16 for Dutch with a Papiamento insertion.

(15) “*Después es de* **bloem** *negro*”(Spanish ML, Dutch insertion)
   Next is the flower black
   “Next is the black flower”
(16) “*Onder die rooie* **kas**, **un kurason** *wit*”(Dutch ML, Papiamento insertion)
   Below that red house a heart white
   “Below that red house, a white heart”

This type of insertion occurred 66 out of 1,574 times in our data (37 times for Dutch as the ML and 29 times for Spanish/Papiamento as the ML) and in all cases, the adjective followed the noun. Note that the second example contradicts both the predictions from the MLF and the MP. We would like to suggest the preference for the postnominal adjective in these cases may be explained from the perspective of processing economy. If the adjective would precede the noun, the speaker would have to switch back and forth between languages several times: the verb in the ML, the determiner in the inserted language, then the adjective in the inserted language and the noun in the ML again, which may not be the most economic strategy.

In addition to these determiner-noun insertions, there are some insertion types that seem to be used as specific strategies by individual speakers. For instance, one Spanish heritage speaker uses almost exclusively Dutch (det-)noun-adjective insertions, always with a postnominal adjective, as in example 17:

(17) “*Arriba del* **hartje wit** *hay un* **sleutel groen**”
   Above the heart white there is a key green
   “Above the white heart there is green key”

The same individual variation was found in other insertion types as well. For instance, postnominal adjectives with noun insertions into Dutch were dispreferred by most participants, but for some speakers this was actually the preferred option. This indicates that different participants seem to adhere to different strategies.

In part, these different strategies were related to the specific linguistic communities. For instance, Papiamento speakers of all age groups categorically produced postnominal adjectives when the ML was Papiamento, whereas the Spanish speaking participants showed variation in the same context. However, this variation mostly pertained to the younger participants; the adult Spanish speakers almost categorically preferred noun-adjective order, similarly to the Papiamento speakers. Interestingly, the reversed pattern was observed when Dutch was the matrix language: here, an age effect was observed for the Papiamento speakers, but not the Spanish speakers. While Papiamento speaking children preferred noun-adjective word order, the adults almost categorically produced prenominal adjectives. This difference between children and adults is in line with studies by Vihman (2018) and Urbaneja (2020), although the former was a case study of two children and the second did not compare children and adults from the same community. Two studies that have compared children and adults from the same community (Balam et al., 2021; Phillips and Deuchar, 2021) did not find any differences between the two age groups. This topic needs to be investigated further in future studies.

The difference between the Papiamento and the Spanish speakers in our study is most likely not related to linguistic differences between Papiamento and Spanish, given that the two languages overlap in terms of word order in the nominal domain. However, there are important sociolinguistic and sociohistorical differences between these communities that may explain their differential behavior. First, the Papiamento community in the Netherlands is bigger and more established, compared to the migrant Spanish community, in part because it has a longer history of post-colonial relationship. This may mean there is more contact between the members of the Papiamento community than between Spanish-speaking immigrants and their descendents. Second, all Papiamento HSs, even those who were born in Aruba or Curaçao had knowledge of Dutch before migration given the official stats of Dutch, contrary to Spanish HSs who were born in Spanish-speaking countries. Therefore, it is possible that language mixing is more common in the Papiamento community, and that for this reason there are clearer community norms than for our Spanish-speaking participants. In fact, we know from previous research (Muysken et al., 1996; Parafita Couto and Gullberg, 2019) that Papiamento speakers in the Netherlands have clear norms when it comes to the directionality of code-switching: they tend to use Papiamento as the matrix language and insert Dutch elements. This may explain why, in this direction of code-switching, Papiamento-speaking children converge on the adult pattern from an early age, as they are exposed to this type of switches relatively more often and from an early age onward.^5^ The opposite direction—inserting nouns from the HL into Dutch—is less common in the Papiamento community, which may explain why children take more time to converge on the adult-like adjective-noun word order. In fact, Papiamento-Dutch bilingual children start out preferring the opposite word order—noun adjective—during childhood and, to some extent, still produce it during the teenage years. It is not until adulthood that they converge on what seems to be the target pattern in their community.

The Spanish-speaking differs from the Papiamento-speaking group in several ways. First of all, while the adult participants categorically prefer noun-adjective order when Spanish is the ML, similar to the Papiamento speakers, Spanish-speaking children and teens show more variability in this direction than their Papiamento-speaking counterparts. It may be the case that these speakers are less accustomed to code-switching in general, and as a result of this, children need more time and exposure to code-switching in the input to converge on the adult norm. The two groups also differ in the other code-switching direction: Dutch as the ML with HL words inserted. While the Papiamento speakers categorically choose adjective-noun order in this direction, all Spanish-speaking age groups, including the adults, show a considerable degree of variation. The increased variability in this code-switching direction may indicate that they are less accustomed to this direction, and therefore no clear-cut norms have been established. Given that we do not have information on the code-switching habits for our Spanish-speaking participants, these explanations remain rather speculative and need to be substantiated by further research.

In sum, our data suggest that word order variation in code-switched constructions in the nominal domain is determined by various factors, both linguistic ones (the matrix language and/or the language of the adjective, the type of insertion) and extra-linguistic ones (community and age group). Therefore, the field needs to broaden its focus and take into account all the different variables that may play a role, either by careful controlling of the materials and/or the participants, or by including many variables as potential predictors, which is the approach taken in this study. We contend that, while theories such as the MLF or the MP have been essential in our understanding of code-switching, we also need to acknowledge that any theory that focuses on purely grammatical factors probably cannot be considered an accurate reflection of what happens in reality. As our study, as well as other recent studies (*cf.* Parafita Couto et al., 2021) demonstrate, the reality of code-switching is too complex to reduce it to a single variable. We would like to propose that, rather than talking in terms of pure grammatical “constraints” on code-switching, we may need to talk about a set of predictors that can have different weights, and it is our challenge as researchers to identify which predictors should be included in this set and to estimate their relative weights. This aligns with the proposal of Muysken (2013) for modeling and interpreting language contact phenomena, with speakers’ bilingual strategies in specific scenarios of language contact as the starting point. Musyken claims that bilingual strategies are conditioned by social factors, processing constraints of speakers’ bilingual competence, and perceived language distance. As such, the different outcomes should correspond to different interactions of these strategies in bilingual speakers and their communities and more attention should be paid to the links between these strategies and factors.

Finally, we need to acknowledge that our study has some limitations that may have affected our results. Given that adjectives do not occur often in spontaneous speech (Parafita Couto and Gullberg, 2019), and even in semi-spontaneous elicited production (Parafita Couto et al., 2015 found similar patterns using a toy task), we applied a method to specifically elicit them. While this method was successful in eliciting adjectives, it may have made the task less natural. From literature on spontaneous oral production, we know that speakers usually do not frequently switch between the noun and the adjective (Parafita Couto and Gullberg, 2019). When adjectives are produced, they usually form an island with the noun, that is, the noun and adjective are inserted together. Conversely, in our data, the vast majority consisted of noun insertions or determiner-noun insertions. This is a clear consequence of the nature of our task: people were explicitly instructed to only name the object in the other language. The effect of the task on the type of insertion is important given that the type of insertion, in turn, was related to word order as well. A challenge for future studies could lie in finding the right balance between leaving the participants free to switch when they choose to, and at the same time make sure they use adjectives. Another recommendation for future work is that it is crucial to collect information about our participants’ code-switching habits and their general proficiency in both their languages. This information would have been very useful to support some of the claims we make based on our data.

Another issue to consider includes priming whereby one speaker’s code-switching facilitates another speaker’s similar switching (Kootstra et al., 2010; Fricke and Kootstra, 2016). A recent study by Berghoff et al. (2023) focused on code-switching at points of non-shared word order across a bilingual’s two languages. Their study delved into the scope of code-switching priming by investigating whether lexical repetition across target and prime, a factor known to boost structural priming, can increase code-switching at points of word order divergence. They tested Afrikaans–English bilinguals and showed that lexical repetition boosts the priming of code-switching in a non-shared word order. Their findings demonstrate that code-switching in production is therefore affected by a dynamic interplay between factors both language-internal (i.e., word order) and language-external (i.e., priming, and specifically lexical repetition).

The research outlined in the present study constitutes an attempt to keep widening the research perimeter on code-switching. Our (so far preliminary) findings call for further research to be able to establish the theoretical and empirical implications of our findings. Only after studying different and similar language combinations in different contact situations will we be able to arrive at a description of the different dimensions that characterize code-switching and unveil the factors that modulate bilingual grammars.

## Data availability statement

The datasets presented in this study can be found in online repositories. The names of the repository/repositories and accession number(s) can be found in the article/supplementary material.

## Ethics statement

The studies involving human participants were reviewed and approved by Complying with the Ethics Code for linguistic research at the Faculty of Humanities at Leiden University, for this study written informed consent from all participants (including the parents/guardians of the child participants) was obtained prior to their participation. Written informed consent to participate in this study was provided by the participants’ legal guardian/next of kin.

## Author contributions

BO: data curation, statistical analysis, and writing. BS and IB: methodology, data collection, data transcription and coding, and writing. MP: conceptualization, methodology, writing, and funding acquisition. All authors contributed to the article and approved the submitted version.

## Funding

BO acknowledges that this work has received support by the European Union’s Horizon 2020 research and innovation programme under the Marie Skłodowska-Curie grant agreement No. 101024053. MP acknowledges support from the María Zembrano program (funded by the Eureopean Union, #NextGenerationEU) and the Traineeship in the Humanities Program at Leiden University (https://www.student.universiteitleiden.nl/en/vr/humanities/research-traineeship?cf=humanities&cd=latin-american-studies-ma).

## Conflict of interest

The authors declare that the research was conducted in the absence of any commercial or financial relationships that could be construed as a potential conflict of interest.

## Publisher’s note

All claims expressed in this article are solely those of the authors and do not necessarily represent those of their affiliated organizations, or those of the publisher, the editors and the reviewers. Any product that may be evaluated in this article, or claim that may be made by its manufacturer, is not guaranteed or endorsed by the publisher.

## Footnotes

^1^The Dutch Central Bureau of Statistics (CBS) reports this number for migrants from the Dutch Antilles, which also include the English-speaking islands of Sint-Maarten, Sint-Eustatius and Saba.

^2^In this and all other examples containing code-switching, the matrix language is in italics and the inserted element in bold font.

^3^Some participants used non-standard gender agreement in unilingual Spanish or Dutch utterances, which is discussed in both Boers et al., 2020 and van Osch et al., 2022. Gender assignment in code-switched nominal constructions is discussed in these publications as well.

^4^Finally, there was an interesting relation between word order and gender in Dutch, which is discussed in van Osch et al. (2022). In Dutch, attributive adjectives are inflected for common nouns and uninflected for neuter nouns, but when used predicatively, the adjective is uninflected for both common and neuter gender. van Osch et al. (2022), which is based on the same dataset as the present paper, but focusing on gender, show that whenever the Dutch adjective is placed after the noun, it is uninflected, which seems to suggest that it may be used as a predicative adjective rather than an attributive one.

^5^In addition, there are many Dutch borrowings in Papiamento (e.g., *stòfzùiger* "vacuum cleaner" < Du. *stofzuiger*). In nominal constructions with an adjective, the adjective follows the noun, i.e., the default word order in Papiamento (e.g., *e **stòfzùiger** pretu* "the black vacuum cleaner" < Du. *stofzuiger*), which is similar to the code-switching pattern found in our data (e.g., *e **huis** pretu* "the black house"). The complete convergence toward noun-adjective word order when inserting Dutch words into Papiamento may be reinforced by the large amount of Dutch borrowings in Papiamento (*cf.* Muysken et al., 1996).
